# Augmenting Rice Defenses: Exogenous Calcium Elevates GABA Levels Against WBPH Infestation

**DOI:** 10.3390/antiox13111321

**Published:** 2024-10-30

**Authors:** Rahmatullah Jan, Sajjad Asaf, Lubna, Muhammad Farooq, Saleem Asif, Zakirullah Khan, Jae-Ryoung Park, Eun-Gyeong Kim, Yoon-Hee Jang, Kyung-Min Kim

**Affiliations:** 1 Department of Applied Biosciences, Graduate School, Kyungpook National University, Daegu 41566, Republic of Koreazakirullah371@gmail.com (Z.K.); 2 Coastal Agriculture Research Institute, Kyungpook National University, Daegu 41566, Republic of Korea; 3 Natural and Medical Science Research Center, University of Nizwa, Nizwa 616, Oman; sajadasif2000@gmail.com (S.A.);; 4 Department of Agriculture Biology, College of Agriculture and Life Sciences, Jeonbuk National University, Jeonju 54896, Republic of Korea; 5 Crop Breeding Division, National Institute of Crop Science, Rural Development Administration, Wanju 55365, Republic of Korea; 6 National Agrobiodiversity Center, National Institute of Agricultural Sciences, Rural Development Administration, Jeonju 54874, Republic of Korea; 7 Gene Engineering Division, National Institute of Agricultural Sciences, Rural Development Administration, Jeonju 54874, Republic of Korea

**Keywords:** calcium, gamma-aminobutyric acid, white-backed planthopper, oxidative stress, antioxidant activity, hormones

## Abstract

This study investigates the impact of exogenous calcium and gamma-aminobutyric acid (GABA) supplementation on rice growth and stress tolerance under white-backed planthopper (WBPH) infestation. We evaluated several phenotypic traits, including shoot/root length, leaf width, tiller number, panicle length, and relative water content, alongside physiological markers such as oxidative stress indicators, antioxidant enzymes activities, hormonal levels, and amino acids biosynthesis. Our results indicate that WBPH stress significantly reduces growth parameters but calcium and GABA supplementation markedly enhance shoot length (by 26% and 36%) and root length (by 38% and 64%), respectively, compared to WBPH-infested plants. Both supplementations also reduced oxidative stress, as evidenced by decreased H_2_O_2_ and O_2_^•−^ levels and a lower electrolyte leakage. Notably, calcium and GABA treatments increased antioxidant enzyme activities, with GABA boosting catalase (CAT) activity by 800%, peroxidase (POD) by 144%, and superoxide dismutase (SOD) by 62% under WBPH stress. Additionally, calcium and GABA enhanced the accumulation of stress hormones (abscisic acid ABA) and salicylic acid (SA) and promoted stomatal closure, contributing to improved water conservation. This study reveals that calcium regulates the GABA shunt pathway, significantly increasing GABA and succinate levels in both root and shoot. Furthermore, calcium and GABA supplementation enhance the biosynthesis of key amino acids and improve ion homeostasis, particularly elevating calcium (Ca), iron (Fe), and magnesium (Mg) levels under WBPH stress. Overall, this study highlights the potential of exogenous calcium and GABA as effective strategies for enhancing rice plant tolerance to WBPH infestation by modulating various physiological and biochemical mechanisms. Further research is warranted to fully elucidate the underlying mechanisms of action.

## 1. Introduction

Rice stands as one of the principal food crops globally, serving as a primary food source for more than 50% of the global population. Specifically, Asia accounts for more than 90% of the world’s rice production, yielding a staggering 481 million tons annually, underscoring its pivotal role as a crucial crop in the region [1]. In addition to its economic significance, it contains abundant genetic diversity through numerous traditional varieties and ancestral species. However, diverse challenges, stemming from both living organisms and environmental factors, significantly constrain its productivity on a global scale [2]. The warm and humid climatic conditions prevalent in rice cultivation areas create favorable environments for the proliferation of insects, with over 100 known species posing threats to this crop [3]. Out of these, approximately 20 species hold significant economic importance in rice cultivation, with total yield losses due to insect pests ranging from 21% to 51%. Notably, planthoppers alone were responsible for an annual yield reduction of one million tons between 1970 and 1990 [4,5]. Among the sucking insect pests affecting rice, economic concerns primarily revolve around planthoppers, with a particular emphasis on the brown planthopper (*Nilaparvata lugens*), white-backed planthopper (*Sogatella furcifera*), and leafhoppers [6]. The white backed planthopper (*Sogatella furcifera*) is distributed across South, South-East, and East Asia [7]. Both the nymphs and adults of the white-backed planthopper suck water and nutrients from plant sap. This feeding behavior results in leaf yellowing, reduced tillering, diminished plant height, and unfilled grains, particularly under high insect populations. Consequently, affected plants exhibit symptoms such as browning, wilting, and eventual death [8].

Rice plants employ a combination of primary and secondary metabolites, along with hormones and amino acids, to facilitate not only growth and development but also to fortify defense mechanisms against a spectrum of abiotic and biotic stresses [9]. Among these compounds, GABA, a four-carbon non-proteinogenic amino acid, holds significant importance across biological domains, playing dual roles as both a metabolite and a signaling molecule in plants [10,11,12]. In addition its functional versatility extends to the maintenance of carbon/nitrogen (C/N) balance and the regulation of plant development [13]. GABA is crucial for plant defense against a range of abiotic and biotic stressors, including low light, salinity, nitrogen starvation, drought, temperature fluctuations, insects, and necrotrophic fungi [14,15]. Research has shown that GABA promotes plant growth, increases biomass, enhances yield traits, and improves cell integrity under stress conditions [16]. Similarly, GABA plays an important role in scavenging reactive oxygen species (ROS) such as H_2_O_2_, O_2_^•−^, reducing malondialdehyde (MDA) contents, and minimizing electrolyte leakage, thereby enhancing the plant antioxidant system during stress conditions [17]. Additionally, GABA induces the production of phytohormones such as benzoic acid (BA), ABA, salicylic acid (SA), and indole acetic acid (IAA) when plants are exposed to stress conditions [18]. GABA plays a crucial role in mitigating plant stress by modulating the expression of GABA shunt pathway genes, such as *GAD*, *GABA-T*, and *SSADH* (Succinic semialdehyde dehydrogenase), in response to a wide range of abiotic and biotic stressors [19]. GAD catalyzes the conversion of glutamate to GABA, while GABA-T facilitates the transformation of GABA into succinic semialdehyde. Subsequently, SSADH converts succinic semialdehyde into succinic acid. This pathway produces NADH (nicotinamide adenine dinucleotide hydrogen), providing energy and a carbon skeleton to the TCA cycle, thereby regulating redox reactions under oxidative stress [20,21]. Additionally, GABA contributes to the regulation of free amino acids (FAAs), essential nutrients, cell integrity, and antioxidant activity during stress conditions [22].

Calcium is widely recognized for its pivotal role in plant growth and development [23]. A recent review article demonstrated that calcium application enhanced seven abiotic stress tolerances, such as drought, salt, flooding, low temperature, high temperature, acid rain, and heavy metal stresses [24]. The function of exogenous calcium against biotic stress is not well documented; however, it was recently reported that its application increases resilience to *Botryosphaeria dothidea* via activation of autophagy and salicylic acid (SA) [25]. It was also reported that calcium activates the Calmodulin-binding transcription activators (CAMTA3) protein by binding to calmoduline, which results in SA regulation, and controls the *Arabidopsis thaliana* defense responses against pathogens [26]. It regulates key cellular processes including cytoplasmic streaming, thigmotropism, gravitropism, cell division, differentiation, elongation, photomorphogenesis, and cell polarization [27]. Additionally, calcium maintains the structure of the cell wall and cell membrane while actively regulating cellular metabolism under normal and stressed conditions [28]. Notably, it induces the plant’s defense system and increases free cellular calcium levels during both abiotic and biotic stress [29]. Previous research has demonstrated that calcium, serving as a cofactor, promotes the accumulation of GABA [30]. Its interaction with calmodulin influences GABA synthesis through the regulation of GAD (glutamate decarboxylase) activity [31]. Therefore, this study aims to investigate the effect of exogenous application of calcium and GABA on rice plant under WBPH infestation.

## 2. Materials and Methods

### 2.1. Material Used and Experimental Design

In this study, we used Ilmi rice cultivar provided by Kyung-Min Kim (Plant Molecular Breeding Lab, Kyungpook National University, Republic of Korea). We used five treatments group in this study: the first group was control plants (Cont), the second was WBPH-infested rice plants (WBPH), the third was WBPH-infested plants supplemented with calcium (Ca + WBPH), the fourth group was WBPH-infested plants supplemented with GABA (GABA + WBPH), and the fifth group was only GABA-supplemented plants (GABA). Initially, the seeds were surface-sterilized with fungicides and then incubated in the dark at 33 °C for three days [32]. Following successful germination, the seedlings were transferred to pots and maintained in the dark for three days. After three days in dark conditions, the seedlings were transferred into pots. The experiment was conducted in pots in a greenhouse using three biological replicates. The following greenhouse conditions were followed (28–30 °C, 60% humidity, and a 16/8 h photoperiod) as mentioned previously [33]. Initially, we screened the growth of the rice plants on different concentrations of calcium (2, 4, 6, 8, and 10 µM) (Supplementary Figure S1). On the basis of this screening, we selected 4 µM of calcium and treated one-month-old plants (one month after transfer into pots). The GABA group plants were treated with 15 mM of GABA mixed in water and applied as a solution directly. On the basis of our previous study, we selected a 15 mM concentration of GABA [34]. Both the calcium and GABA were treated one week before the WBPH infestation. Plants were exposed to WBPH in an insectarium, and phenotypic assessments were conducted at the final stage of growth. The plants treated with WBPH were kept separately in the insectarium and infested with 100 WBPH per plant before the GABA/calcium treatment. Plant shoot length, root length, leaf width, tiller number, panicle length, and number of seeds per panicle were evaluated at the last stage of plant growth with continued exposure to WBPH, GABA, and calcium.

### 2.2. DAB and Trypan Blue Histochemical Staining and Quantification of H_2_O_2_ and O_2_^•−^ and Electrolyte Leakage

In situ staining for hydrogen peroxide (H_2_O_2_) was conducted using 3,3-diaminobenzidine (DAB) solution, following the protocol outlined by [35]. In summary, leaves were harvested one week after infestation or calcium/GABA treatment, directly submerged in DAB solution, and incubated at 27 °C for 24 h [36]. The stained leaves were decolorized by boiling in 95% ethanol until the brown spots became distinctly visible. After cooling, the leaves were immersed in a solution of lactic acid, phenol, and water in equal volumes (1:1:1, *v*/*v*/*v*) and subsequently photographed. For in situ staining of dead cells, leaves were collected after one week of infestation or calcium/GABA treatment, immersed in trypan blue solution, and incubated at 25 °C for 6 h. The leaves were then decolorized by boiling in 95% ethanol, transferred to the lactic acid, phenol, and water solution (1:1:1, *v*/*v*/*v*), and immediately photographed.

H_2_O_2_ level was evaluated according to the method outlined by [37]. Briefly, 0.1 g of leaf sample was homogenized and extracted with 5 mL of 0.1% trichloroacetic acid (TCA) solution. The extract was then centrifuged at 12,000× *g* for 15 min, after which 0.5 mL of the supernatant was collected. The absorbance at 390 nm was recorded following the combination of 1 mL of 1 M potassium iodide with 0.5 mL of 10 mM phosphate buffer (pH 7.0).

Superoxide anion production was quantified following the protocol outlined in [38]. Fresh leaf samples (200 mg) were ground in liquid nitrogen and homogenized in a 100 mM sodium–phosphate buffer containing 1 mM diethyl dithiocarbamate. Following centrifugation, the supernatant was assessed for superoxide anion levels by measuring its capacity to reduce NBT in a buffered solution, with absorbance readings recorded at 540 nm.

To assess electrolyte leakage, leaf samples (100 mg) were cut into 5 mm pieces and submerged in 10 mL of distilled deionized water in sealed test tubes. After 2 h at 32 °C, initial electrical conductivity (EC1) was measured using a conductivity meter (CM-115, Kyoto Electronics, Kyoto, Japan). Samples were then autoclaved at 121 °C for 20 min to ensure complete tissue breakdown and electrolyte release. After cooling to 25 °C, final conductivity (EC2) was measured. Electrolyte leakage (EL) was measured using the formula $EL=\frac{EC1}{EC2}\times 100$, expressing the percentage of electrolytes released from the leaf tissues.

### 2.3. Measurement of CAT, POD, SOD, and MDA

For CAT, POD, SOD, and MDA content analysis, fresh leaves were collected randomly after one week of WBPH infestation or calcium/GABA treatment. To measure the catalase activity, the protocol optimized by LH Johansson and LAH Borg (1988) was followed [39,40]. Briefly, fresh leaf tissue of 200 mg were collected and ground in liquid nitrogen and mixed with 200 µL methanol. The extracted crude catalase was mixed with 0.5 mL of 0.2 mM phosphate buffer (H_2_O_2_ and KH_2_PO_4_-NaOH) (pH 7.0). The activity of catalase enzyme was measured by observing the reduction in absorbance levels of hydrogen peroxide at a wavelength of 240 nm. A single unit of catalase (CAT) was established as the quantity of hydrogen peroxide broken down per minute for each milligram of protein.

To measure POD activity, assay mixture was prepared by mixing 125 µL phosphate buffer, 50 µM pyrigallol, 50 µM H_2_O_2_, and 100 µL of enzyme extract. The reaction was kept at 25 °C for 5 min. After incubation, 0.5 mL of 5% H_2_SO_4_ was added to terminate the reaction. The absorbance of the samples was measured at 420 nm and the POD activity was expressed in terms of enzyme unit per gram.

The concentration of SOD was determined using a method inspired by the procedure outlined by Lubna et al. in 2022 [41]. Briefly, the leaves were ground in liquid nitrogen and homogenized in the buffer solution (1.5 mL) containing 50 mM Tris-HCl and 10 mM EDTA at pH of 8.0. Following this, a 100 µL aliquot of the resulting extract was combined with 100 µL of pyrogallol (7.5 mM) and kept at 25 °C for 10 min. The reaction was terminated by adding 50 µL of 1N HCl. The absorbance of the resulting solution was measured at a wavelength of 420 nm. The SOD activity was measured as a percentage by using the following formula:

SOD activity (%)=[(1−(A−B)/C)]×100

where A represents the absorbance of the sample with added pyrogllol, B is the absorbance of the sample without pyrogallol, and C is the absorbance of the control, which is a buffer solution with added pyrogallol. SOD activity was expressed in terms of enzyme unit per gram.

MDA levels were determined using a lipid peroxidation kit from Sigma Korea, following the detailed protocol outlined in our previous study [42].

### 2.4. ABA and SA Quantification

After one week of stress exposure or calcium/GABA treatment, levels of abscisic acid (ABA) and salicylic acid (SA) were quantified. Leaf samples were collected following a week of WBPH infestation, then freeze-dried to isolate the SA and ABA. The dried samples were ground into a fine powder using liquid nitrogen, and both SA and ABA were extracted and quantified using the Sialic Acid (SA) ELISA Kit and the Plant Abscisic Acid ELISA Kit from LifeSpan BioSciences (2401 Fourth Avenue, Suite 900, Seattle, WA, USA). Quantification was carried out according to the methods outlined in the user manuals.

### 2.5. GABA Extraction

To extract and quantify GABA, leaves and roots of three plants were collected randomly after one week of WBPH infestation or calcium/GABA treatment, following the method outlined by [43]. To extract GABA, 300 mg of leaves and roots were ground in liquid nitrogen and then mixed in 2 mL of chilled solvent composed of methanol, chloroform, and water in a 5:2:1 ratio (*v*/*v*/*v*). The homogenate was stored at −20 °C overnight. The mixture was then shaken on an ice-cold shaker for 30 min and subsequently centrifuged at 12,000 rpm for 10 min. A 1.5 mL aliquot of the supernatant was carefully collected and mixed with 2 mL of deionized water and chloroform in a 2:1 ratio, vortexed thoroughly, and centrifuged at 12,000 rpm for 2 min. The upper phase was then vacuum-dried. GABA was further purified from the dried samples using the method described by [44]. Briefly, each dried sample was treated with 100 µL of acetonitrile and methyl tert-butyldimethylsilyl trifluoroacetamide, heated at 70 °C for 30 min, and 1 µL of the resulting solution was analyzed by gas chromatography using a GC Model 7890 A (Agilent Technologies, Santa Clara, CA, USA) with a BP-5 capillary column. The injector and detector temperatures were maintained at 280 °C, while the oven temperature was initially set to 70 °C for 2 min and then increased at a rate of 5 °C per minute until it reached 300 °C.

### 2.6. Succinate Quantification

Succinate levels were quantified in leaf samples collected one week after WBPH infestation or calcium/GABA treatment, using the Succinate Colorimetric Assay Kit (Sigma-Aldrich, Spruce Street, St. Louis, MO, USA), following the manufacturer’s instructions. Briefly, 10 mg of ground rice leaf tissue from each treatment group was homogenized on ice in 100 µL of succinate assay buffer, and the mixture was then centrifuged at 10,000× *g* for 5 min. The resulting supernatant was directly transferred to a 96-well plate, and the final volume in each well was adjusted to 50 µL using succinate assay buffer. To ensure accuracy, samples from each treatment group were added to the 96-well plate in five technical replicates. After adding 50 µL of the reaction mix the wells were mixed by pipetting. The plate was then incubated at 37 °C for 30 min in the dark, and absorbance was measured at 450 nm (A450). To generate a standard curve, wells were prepared with 0, 2, 4, 6, 8, and 10 µL of a 1 nmole/µL succinate standard solution, each diluted to a total volume of 50 µL with succinate assay buffer. The absorbance of the blank well was subtracted from all sample readings, and the succinate concentration (C) was calculated using the following formula:

$$C=\frac{Sa}{Sv}\times 118.09$$

where C represents the final concentration of succinate, Sa is the amount of succinate added to the well, Sv is the volume of the sample in the well, and 118.09 is the molecular weight of succinate.

### 2.7. Iron, Magnesium, and Calcium Ion Evaluation

To assess the accumulation of iron (Fe+), magnesium (Mg+), and calcium (Ca^2+^) ions, leaf samples were collected one week after WBPH infestation or calcium/GABA treatment, and immediately freeze-dried. Approximately 0.5 g of the lyophilized sample was ground in liquid nitrogen and homogenized in 7 mL of 65% nitric acid (HNO_3_) combined with 1 mL of 30% hydrogen peroxide (H_2_O_2_). The mixture was then subjected to microwave digestion for 20 min at 180 °C and subsequently cooled for 30 min, as described previously [45]. The resulting solution was analyzed for ion content using inductively coupled plasma mass spectrometry (ICP-MS; Optima 7900DV, Perkin-Elmer, Waltham, MA, USA).

### 2.8. Free Amino Acid Quantification

One week after WBPH infestation or calcium/GABA treatment, free amino acids were quantified using fresh leaves collected randomly. About 500 mg of fresh leaf tissue was ground in liquid nitrogen and then homogenized in 10 mL of 70% methanol. The homogenate was shaken at room temperature for 24 h. The levels of free amino acids were then assessed using the EZ: faast amino acid analysis kit (Phenomenex, Santa Clara, CA, USA), following the manufacturer’s instructions. The amino acid content was subsequently analyzed by GC-MS using a Hewlett-Packard 6890N/5975 system (Agilent Technologies, Torrance, CA, USA) equipped with a ZB-AAA amino acid analysis column (10 m × 0.25 mm). The analysis was carried out with a constant carrier gas flow and an oven temperature program as outlined by [46].

### 2.9. Chlorophyll and Relative Water Content

To evaluate the total chlorophyll content, a Soil–Plant Analysis Development (SPAD-502 plus; Konica Minolta Sensing, Seoul, Republic of Korea) meter was utilized. For each measurement, three leaves were randomly selected in triplicate after one week of WBPH infestation or calcium/GABA treatment. To measure the relative water content (RWC), ten leaves were systematically collected from each experimental group after one week of stress exposure or calcium/GABA treatment. The fresh weight (FW) of the leaves was immediately recorded upon harvest. The leaves were then submerged in distilled water for 4 h to obtain their turgid weight (TW). Following this, the leaves were dried in an oven at 80 °C for 24 h to determine their dry weight (DW). The relative water content was calculated using the following formula:

$$RWC=\frac{FW-DW}{TW-DW}\times 100$$

In this formula, FW is the fresh weight, TW is the turgid weight, and DW is the leaf dry weight. This experiment was repeated three times.

### 2.10. Statistical Analysis

All data were analyzed using GraphPad Prism software (version 5.01; GraphPad, San Diego, CA, USA). A one-way analysis of variance (ANOVA) was employed to evaluate the dataset, which included three independent biological replicates. Mean values were compared using Bonferroni post hoc tests. Statistical significance was indicated by the following thresholds: * p < 0.05, ** p < 0.01, and *** p < 0.001.

## 3. Results

### 3.1. Exogenous Calcium and GABA Promote Rice Growth Under WBPH Stress

**Figure 1:**
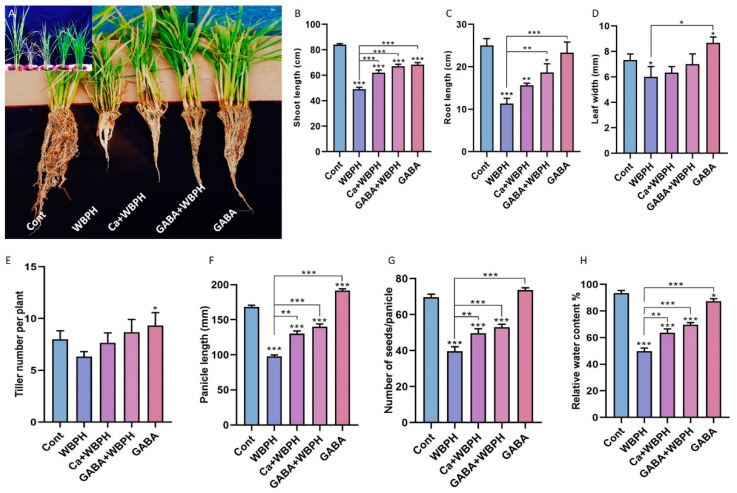
Calcium and GABA enhance rice growth parameters against WBPH stress. Panel (**A**) provides a pictorial representation of plant length and development. Panels (**B**–**H**) depict the effects of calcium and GABA supplementation on shoot length, root length, leaf width, tiller number, panicle length, seed number per panicle, and leaf relative water content, respectively. Data in panels (**B**–**H**) are derived from three independent biological replicates, presented as means ± standard deviation (SD), and statistically analyzed using Bonferroni post hoc tests. Statistical significance is denoted by * (*p* < 0.05), ** (*p* < 0.01), and *** (*p* < 0.001).

Here, we examined the growth pattern of rice plants under WBPH infestation, with particular focus on the effects of calcium and GABA supplementation. Our evaluation encompassed several phenotypic traits, including shoot/root length, leaf width, tiller number, panicle length, and relative water content (Figure 1). Our data revealed distinct responses in rice plants treated with calcium and GABA under WBPH stress, as compared to non-treated plants. WBPH stress generally resulted in a reduction in almost all evaluated phenotypic traits. However, supplementation with calcium and GABA significantly enhanced these traits compared to non-treated infested plants.

Specifically, the shoot length in both calcium-treated and GABA-treated plants under WBPH stress (Ca + WBPH and GABA + WBPH) increased 26% and 36%, respectively, compared to WBPH-infested plants (Figure 1B). Similarly, root length in Ca + WBPH and GABA + WBPH plants increased 38 and 64%, respectively, compared to WBPH-infested plants (Figure 1C). Contrarily, the leaf width and tiller number did not show significant enhancement with calcium and GABA application under WBPH stress (Figure 1D,E). However, panicle length increased 33 and 43%, seed number per tiller increased 25 and 33%, and water relative contents increased by 27 and 39% in Ca + WBPH and GABA + WBPH plants, respectively, compared to WBPH-infested plants (Figure 1F–H). Interestingly, we observed that GABA application in non-infested plants significantly increased leaf width, tiller number, and panicle length compared to control plants. Overall, these results suggest that the application of calcium and GABA can significantly promote growth parameters in rice plants under WBPH infestation. This underscores the potential of these supplements as effective strategies against WBPH stress in rice cultivation.

### 3.2. Calcium and GABA Are Key Factors in Boosting WBPH Stress Tolerance

**Figure 2:**
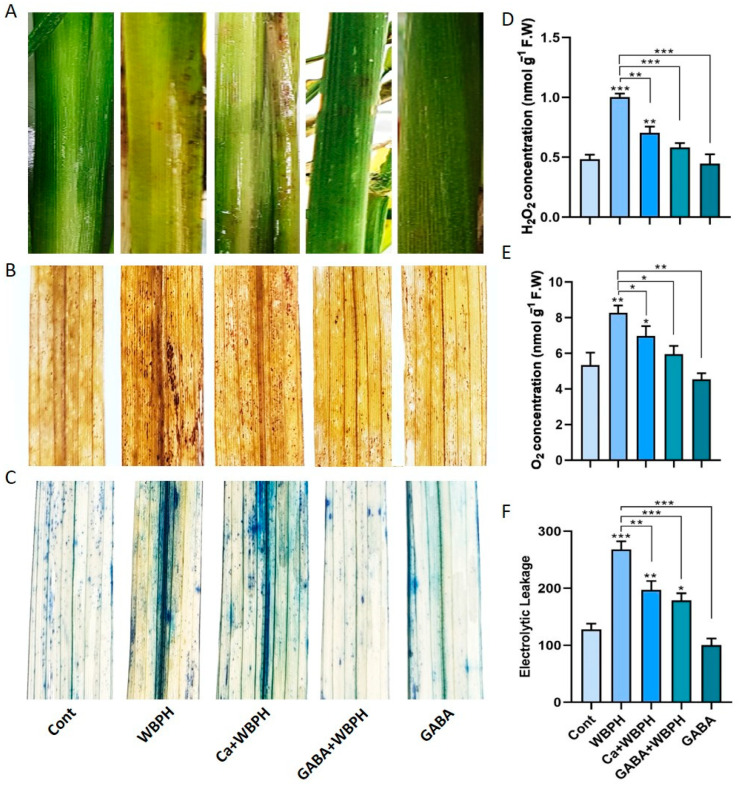
Exogenous calcium and GABA mitigate WBPH-induced oxidative stress in rice plants. Panel (**A**) illustrates symptoms of WBPH infestation on rice stems at the seedling stage. Panels (**B**,**C**) depict the in situ detection of oxidative damage in rice leaves induced by the generation of reactive oxygen species, with (**B**) showing DAB staining and (**C**) showing trypan blue staining. Panel (**D**) shows H_2_O_2_ accumulation, while panel (**E**) displays O_2_^•−^ accumulation in rice leaves under different treatments. Panel (**F**) represents electrolytic leakage from rice leaves under various treatments. Data presented in panels (**D**–**F**) were obtained from three independent biological replicates (± standard deviation, SD), and statistical analysis was conducted using Bonferroni post hoc tests. Significance levels are denoted by * (*p* < 0.05), ** (*p* < 0.01), and *** (*p* < 0.001).

Our study further explored the role of exogenous calcium and GABA in enhancing the stress tolerance of plants infested with WBPH. The focus was on mitigating oxidative stress caused by WBPH infestation. We assessed the level of oxidative stress by examining the accumulation of key indicators such as H_2_O_2_ and O_2_^•−^ and electrolytic leakage. These indicators were visualized using DAB staining techniques and quantitative analysis. The results, as depicted in Figure 2A, showed the plants infested with WBPH were more susceptible to damage and stress. This susceptibility was reduced in plants treated with calcium and GABA (Ca + WBPH and GABA + WBPH). This trend was consistent with the results obtained from DAB and trypan staining (Figure 2B,C).

Quantitative analysis of H_2_O_2_ and O_2_^•−^ and electrolytic leakage revealed a significant reduction in these parameters when calcium and GABA were applied under WBPH stress, compared to plants only infested with WBPH (Figure 2D–F). Specifically, H_2_O_2_ and O_2_^•−^ levels were reduced by 29 and 42% and 15 and 28%, respectively, in Ca + WBPH and GABA + WBPH plants, compared to WBPH-infested plants. Similarly, electrolytic leakage was reduced by 26 and 33% in Ca + WBPH and GABA + WBPH plants, respectively, compared to WBPH-infested plants. Interestingly, despite the reduction, the levels of H_2_O_2_ and O_2_^•−^ and electrolytic leakage were still significantly higher in WBPH-infested, Ca + WBPH, and GABA + WBPH plants compared to control plants. This indicates that while WBPH infestation induces oxidative stress, the application of calcium and GABA can mitigate this stress, but not completely eliminate it. These findings suggest that exogenous calcium and GABA can enhance the tolerance of plants to WBPH infestation by reducing the oxidative stress. However, further research is needed to fully understand the underling mechanisms.

### 3.3. Calcium and GABA Mitigate WBPH Stress Through Lipid Peroxidation Reduction and Antioxidant Activity Enhancement

**Figure 3:**
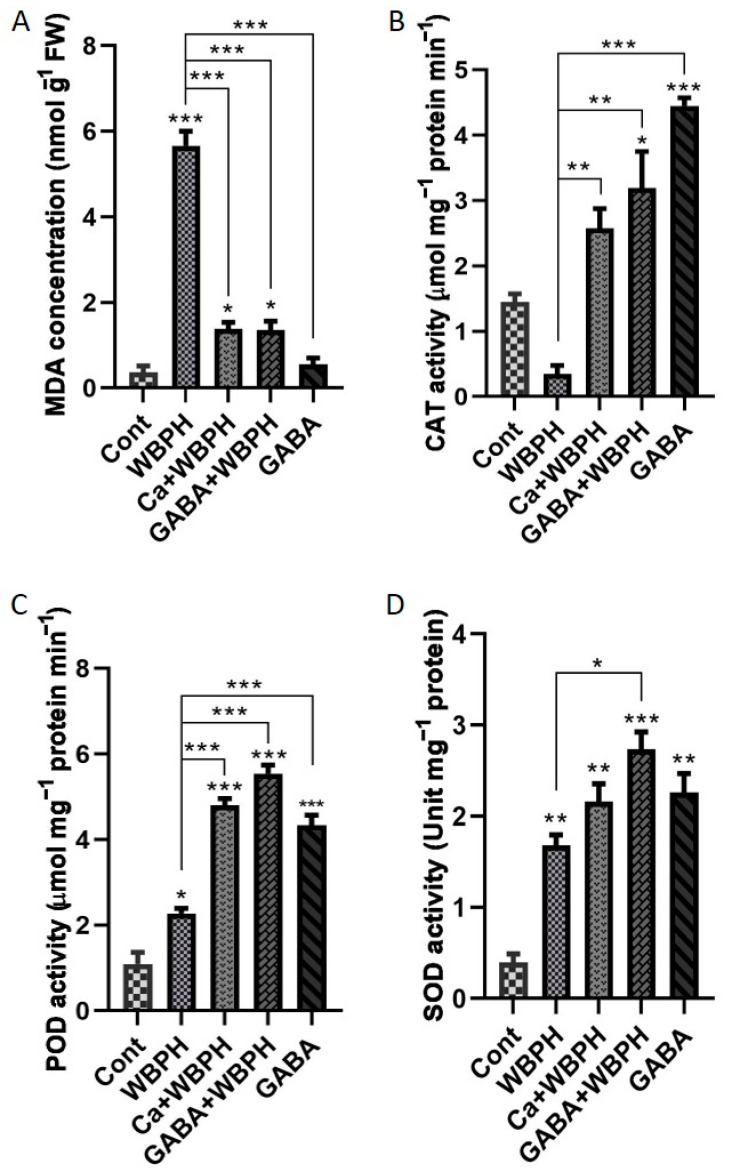
Calcium and GABA supplementation mitigate lipid peroxidation and enhance antioxidant enzymatic activity in rice plants under WBPH stress. Panel (**A**) illustrates lipid peroxidation, measured by MDA (malondialdehyde) contents accumulation under different treatments in response to WBPH stress. Panels (**B**–**D**) depict the activities of catalase, peroxidase, and superoxide dismutase enzymes, respectively. Data were derived from three independent biological replicates (± standard deviation, SD), and statistical significance was assessed using Bonferroni post hoc tests. Significance levels are denoted by * (*p* < 0.05), ** (*p* < 0.01), and *** (*p* < 0.001).

In our research, we conducted a comprehensive examination of the oxidative stress response in plants subjected to WBPH stress. We focused on the activities of CAT, POD, and SOD, as well as membrane lipid peroxidation (Figure 3). These elements serve as key indicators, shedding light on the level of oxidative damage plants endure under stressful conditions. Our findings showed a notable increase in malondialdehyde (MDA) levels in plants under WBPH stress, both with and without calcium (Ca + WBPH) and GABA (GABA + WBPH) treatments, when compared to the control group. However, the MDA content in Ca + WBPH and GABA + WBPH plants was significantly lower by 75% and 76%, respectively, than in WBPH-infested plants (Figure 3A). We observed that the decrease in MDA content corresponded with the accumulation patterns of H_2_O_2_ and O_2_^•−^ accumulation (Figure 2D,E). Non-treated plants exhibited a clear increase in MDA content, which was higher than the levels in plants treated with calcium and GABA under WBPH stress.

Simultaneously, we noticed distinct accumulation patterns of CAT, POD, and SOD activities in treated and non-treated plants compared to the control group (Figure 3B–D). The application of calcium and GABA resulted in a significant increase in CAT activity by 600% and 800%, respectively, under WBPH stress compared to WBPH-infested plants. Similarly, POD activity increased by 112% and 144% in Ca + WBPH and GABA + WBPH plants, respectively, compared to WBPH-infested plants. SOD activity increased by 28% and 62% in Ca + WBPH and GABA + WBPH plants, respectively, compared with WBPH-infested plants. Interestingly, applying GABA without WBPH infestation significantly increased the activity of CAT, POD, and SOD compared to the control group. The MDA levels showed an inverse relationship with CAT, POD, and SOD activity in WBPH-infested plants treated with calcium and GABA. This highlights the complex interactions within the plant’s antioxidative defense system. These results provide valuable insights into how plants respond physiologically to WBPH stress and the potential mitigating effects of calcium and GABA treatments.

### 3.4. Calcium and GABA as a Key Regulator of ABA, SA, and Stomatal Dynamics in WBPH Stress

**Figure 4:**
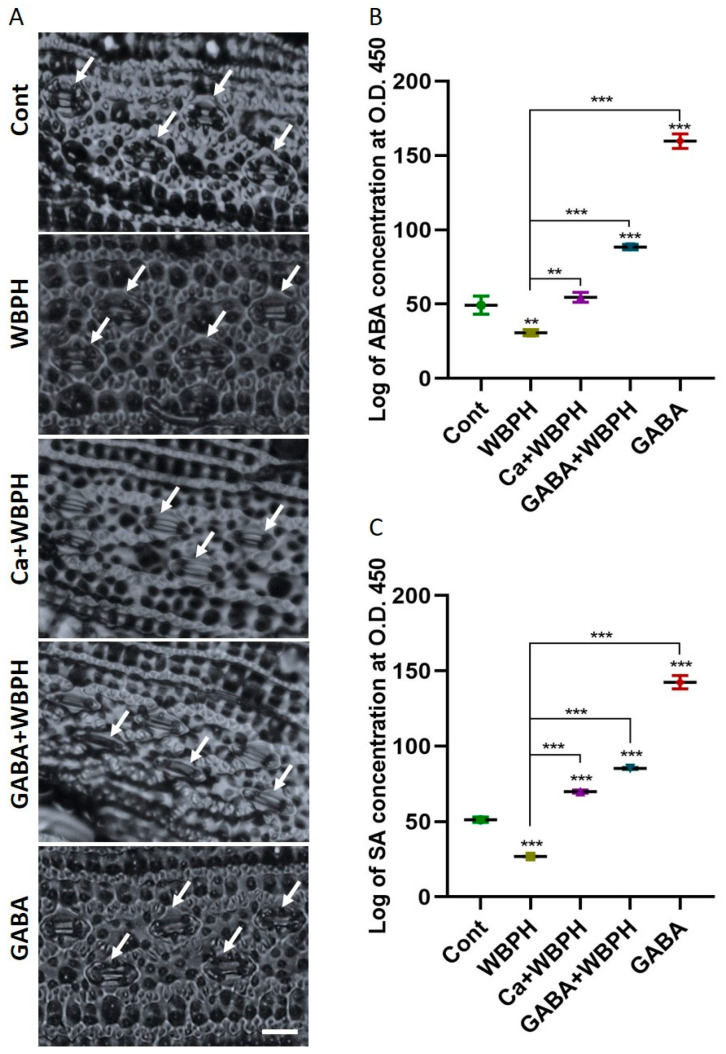
Calcium and GABA induce the synthesis of ABA and SA, facilitating the regulation of stomatal dynamics in rice plants under WBPH stress. (**A**) shows stomata opening and closing patterns induced by calcium and GABA supplementation under WBPH stress. (**B**,**C**) shows accumulation of ABA and SA, respectively, in rice plants induced by calcium and GABA under WBPH stress. The data presented in (**B**,**C**) were analyzed in three independent biological replicates (± standard deviation, SD), and the means were compared using Bonferroni post hoc tests. ** indicates *p* < 0.01, and *** indicates *p* < 0.001. White arrow indicate stomata while scale bare shows 20 µm.

In this research, we explored the accumulation of ABA and SA, and the behavior of stomatal opening under WBPH stress (Figure 4). Both ABA and SA are known to accumulate under various stress conditions, playing a crucial role in plant protection. Our investigation revealed that the application of calcium and GABA promotes stomata closure under WBPH stress, contrasting with the open stomata observed in non-treated WBPH infested plants (Figure 4A). This is significant as the regulation of stomatal opening and closing is a key defense strategy for maintaining water content during stress conditions. We observed that both calcium and GABA significantly increased the levels of ABA and SA under WBPH stress (Figure 4B,C). Specifically, ABA and SA levels were reduced by 37% and 47%, respectively, in WBPH-infested plants compared to control plants. However, in Ca + WBPH and GABA + WBPH plants, the ABA levels increased by 77% and 186%, respectively, compared to non-treated WBPH-infested plants. Similarly, the SA levels increased by 160% and 217% in Ca + WBPH and GABA + WBPH plants, respectively, compared to non-treated WBPH-infested plants. Our results indicate that stomata were closed in Ca + WBPH and GABA + WBPH plants, which also exhibited higher levels of ABA compared to non-treated WBPH-infested plants. This suggests that the application of calcium and GABA enhances ABA accumulation, which in turn promotes stomatal closure, ultimately contributing to water conservation during stress situations. These findings provide valuable insights into the complex interplay between stress hormones, stomatal behavior, and plant defense mechanisms under WBPH stress.

### 3.5. Calcium Enhances GABA and Succinate Accumulation via Regulation of Shunt Pathway Genes Under WBPH Stress

**Figure 5:**
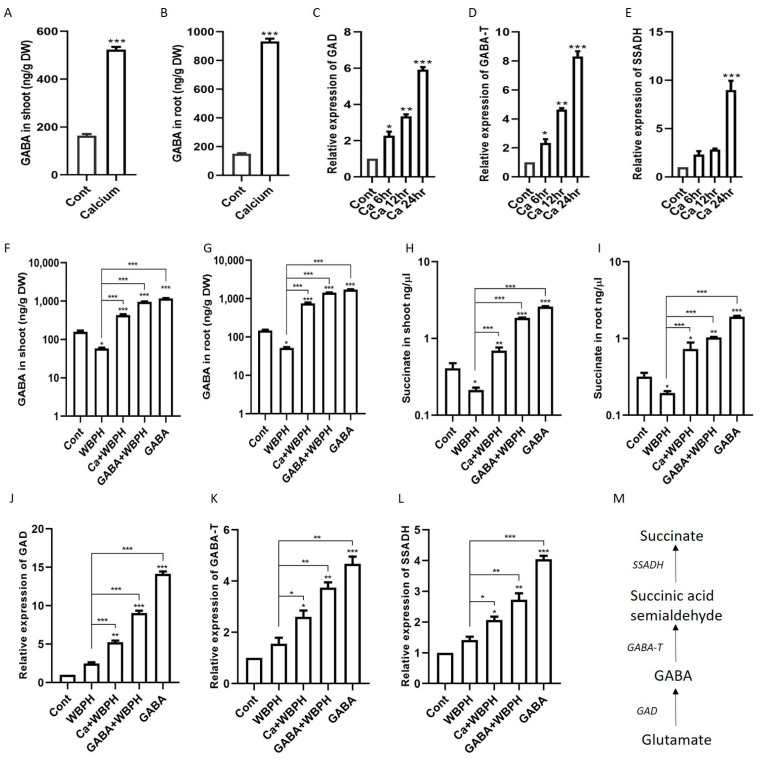
Exogenous calcium triggers the synthesis of GABA and succinate in rice plants, accompanied by the upregulation of their respective biosynthetic genes. Panels (**A**,**B**) show the increased levels of GABA in shoot and root, respectively, following calcium application. Panels (**C**–**E**) display the temporal expression patterns of key biosynthetic genes *GAD*, *GABA-T*, and *SSADH*, after calcium supplementation. Panels (**F**,**G**) depict GABA accumulation in shoot and root under WBPH stress, while panels (**H**,**I**) show succinate accumulation in shoot and root under the same stress condition. Panels (**J**–**L**) illustrate the expression levels of *GAD*, *GABA-T*, and *SSADH* in calcium- and GABA-supplemented rice plants under WBPH stress. Panel (**M**) outlines the GABA shunt pathway. Data represent means from three independent biological replicates (± standard deviation, SD), with statistical significance indicated by * (*p* < 0.05), ** (*p* < 0.01), and *** (*p* < 0.001) determined via Bonferroni post hoc tests.

In our quest to understand the role of calcium in the regulation of GABA and succinate under WBPH stress, we first quantified GABA, succinate, and their biosynthesis genes (*GAD*, *GABA-T*, and *SSADH*) under calcium supplementation. Our results revealed that the exogenous application of calcium to rice plants significantly enhanced the biosynthesis of GABA, succinate, and the expression of their related genes (*GAD*, *GABA-T*, and *SSADH*) compared to control plants (Figure 5A–E). The expression levels of *GAD* and *GABA-T* consistently increased after 6 h, 12 h, and 24 h, while *SSADH* only showed significant enhancement after 24 h. These findings suggest that calcium application can significantly increase GABA and succinate accumulation in rice plants.

Upon further evaluation, we found that under WBPH stress, calcium also increased the accumulation of GABA and succinate in both the root and shoot. Specifically, calcium increased GABA by 1347% and 644% in the root and shoot, respectively, and succinate increased by 274% and 226% in the root and shoot, respectively, under WBPH stress compared to non-treated plants infested with WBPH (Figure 5F–I). Interestingly, the application of GABA also significantly increased indigenous GABA and succinate under WBPH stress when compared to WBPH-infested non-treated plants. The expression pattern of GAD, GABA-T, and SSADH was similar to the pattern followed by GABA and succinate accumulation (Figure 5J–L). The expression of these genes was higher in GABA non-infested plants followed by GABA + WBPH and Ca + WBPH plants. These results indicate that GABA application also induces the expression of these genes, suggesting that an increase in GABA level can mitigate WBPH-induced stress in rice plants.

### 3.6. Calcium and GABA Regulate Free Amino Acids and Ion Homeostasis During WBPH Infestation

**Figure 6:**
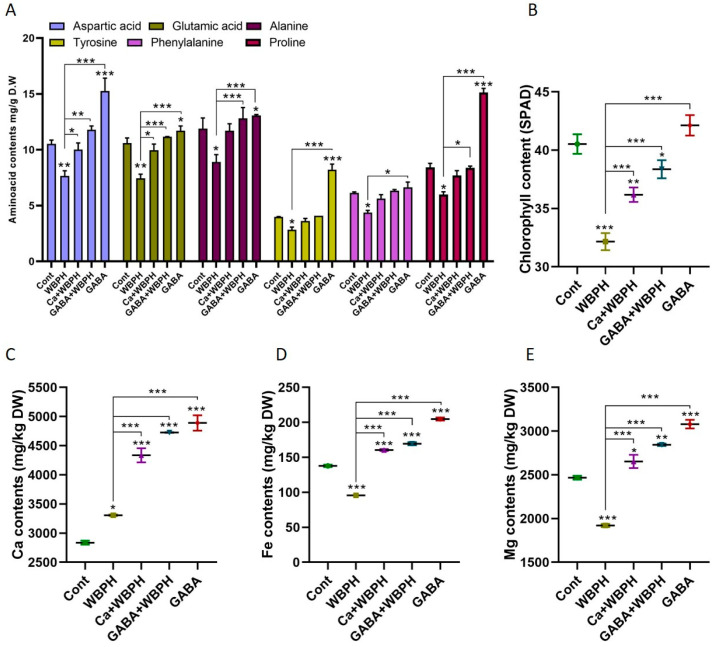
Calcium and GABA supplementation enhance total amino acid content, promote photosynthesis, and regulate Ca, Fe, and Mg ion homeostasis in rice plants exposed to WBPH stress. Panel (**A**) illustrates the regulation of specific free amino acids, panel (**B**) shows chlorophyll content, while panels (**C**–**E**) depict the levels of Ca, Fe, and Mg ions, respectively, under WBPH stress. Data represent means from three independent biological replicates (± standard deviation, SD), with statistical significance indicated by * (*p* < 0.05), ** (*p* < 0.01), and *** (*p* < 0.001) determined via Bonferroni post hoc tests.

Free amino acids play a pivotal role in plants’ defense system against pests. They serve as the primary precursors of plant metabolites involved in the defense mechanism. Our study investigated the biosynthesis patterns of several crucial amino acids (aspartic acid, glutamic acid, alanine, tyrosine, phenylalanine, and proline) under WBPH stress (Figure 6A). Our findings reveal that non-infested plants supplemented with calcium and GABA exhibited a significant increase in the biosynthesis of aspartic acid, glutamic acid, alanine, tyrosine, and proline compared to control plants. This suggests that GABA may enhance the biosynthesis of these amino acids. Interestingly, calcium was found to enhance the biosynthesis of aspartic acid and glutamic acid in WBPH-infested plants compared to non-treated plants. Furthermore, GABA enhanced the biosynthesis of aspartic acid, glutamic acid, alanine, and proline in GABA-WBPH plants compared to WBPH non-treated plants. These results indicate that the application of calcium and GABA can increase the biosynthesis of free amino acids in response to WBPH stress in rice plants. Notably, the chlorophyll content was also significantly enhanced by calcium and GABA application under WBPH stress compared to non-treated plants (Figure 6B).

Further investigation revealed that WBPH stress significantly enhanced calcium ion concentration while reducing iron and magnesium ions compared to control plants (Figure 6C–E). However, the application of calcium and GABA increased the accumulation of calcium ion (31% and 42% in Ca + WBPH and GABA + WBPH, respectively), iron ion (67% and 77% in Ca + WBPH and GABA + WBPH, respectively), and magnesium ion (38% and 48% in Ca + WBPH and GABA + WBPH, respectively) in WBPH-infested plants compared to non-treated WBPH-infested plants. These results provides insights into the role of free amino acids, calcium, and GABA in enhancing plant defense mechanisms against WBPH stress.

## 4. Discussion

**Figure 7:**
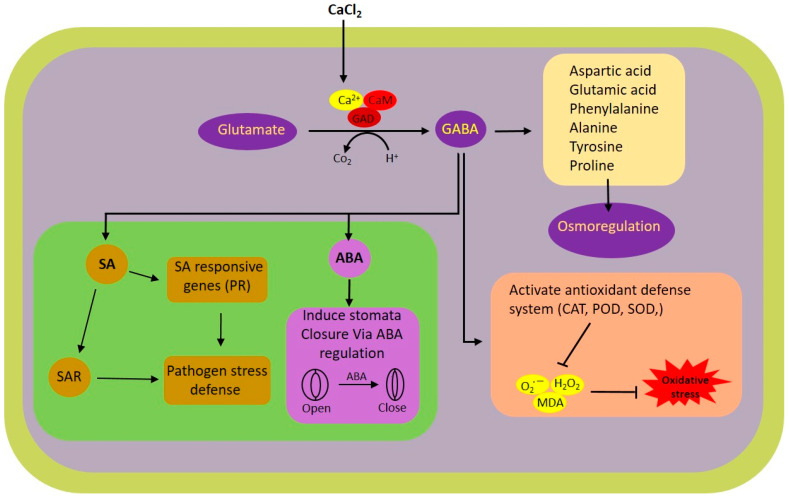
Schematic representation of calcium-induced GABA shunt and its associated pathways regulated during WBPH stress in rice plant. ABA, abscisic acid; Ca^2+^, calcium ion; CaM, calmodulin; CAT, catalyze; GABA, GAD, glutamate decarboxylase; H_2_O_2_, hydrogen peroxide; MDA, malondialdehyde; O_2_^•−^, superoxide radical; POD, peroxidase; PR, pathogenesis-related; SA, salicylic acid; SAR, systemic acquired resistance; SOD, superoxide dismutase.

In this research, we explored the impact of exogenous calcium and GABA supplementation on rice plants subjected to white-backed planthopper (WBPH) stress. The results highlight the substantial potential of calcium and GABA in bolstering the resilience of rice plants to WBPH stress via a range of physiological and biochemical pathways. Notably, the application of calcium was found to modulate the shunt pathway, thereby augmenting GABA synthesis under stress conditions. GABA was instrumental in the modulation of antioxidant activities, mitigating oxidative stress, and regulating stress-responsive hormones in rice plants facing WBPH stress. A schematic representation of the calcium-induced GABA shunt pathway and its associated pathways regulated during WBPH stress in rice plant is illustrated in Figure 7.

Here, we found that the application of calcium regulated the GABA shunt pathway and enhanced the biosynthesis of GABA (Figure 5). The GABA shunt is a crucial biochemical pathway that not only facilitates the synthesis of GABA but also regulates and sustains its levels within the optimal range. This pathway includes three primary enzymatic reactions (Figure 5M) that consist of conversion of glutamate to GABA by the enzyme GAD followed by GABA-T and SSADH which result in GABA biosynthesis in mitochondria [47]. These reactions are essential for both the production and regulation of GABA levels. It has been reported that GABA production is stimulated under stress conditions through two distinct mechanisms: one involves cytosolic acidification leading to the activation of glutamate decarboxylase, and the other is a calcium-dependent activation of the same enzyme [48,49]. Previous studies have indicated that abiotic stresses such as extreme temperatures, salinity, and drought can cause an increase in cytosolic Ca^2+^, which in turn activates calmodulin-dependent glutamate decarboxylase, enhancing GABA synthesis [49]. In line with these observations, our research revealed a significant elevation in Ca^2+^ levels in plants subjected to WBPH stress when compared to the control group (Figure 6C). A study discovered a linkage between herbivore attack and an increase in systemic level of Ca^2+^ in Arabidopsis [50]. This elevation is hypothesized to enhance calmodulin activity, thereby promoting GABA biosynthesis through glutamate decarboxylase activation. It is important to note, however, that our study did not extend to evaluating calmodulin accumulation due to resource constraints, which warrants further investigation to substantiate these findings. Further, our findings revealed a notable reduction in GABA and succinate accumulation in untreated rice plants exposed to WBPH, compared to control plants (Figure 5F–I). However, the application of calcium and GABA significantly bolstered the endogenous levels of GABA and succinate under WBPH stress, surpassing those of untreated infested plants. Examination of gene expression levels for *GAD*, *GABA-T*, and *SSADH* reflected the patterns of GABA and succinate accumulation, further supporting these observations. These results suggest that calcium application can trigger the activation of shunt pathway genes, thereby enhancing GABA accumulation during periods of stress.

The infestation of WBPH induces oxidative stress in rice plants by generating reactive oxygen species (ROS), which leads to membrane damage through lipid peroxidation and an excessive increase in malonaldehyde (MDA) contents. However, our research revealed that GABA significantly mitigated the oxidative stress caused by WBPH infestation. GABA-treated plants exhibited reduced accumulation of H_2_O_2_, O_2_^•−^, and electrolytic leakage compared to non-treated plants under WBPH stress conditions (Figure 2). Symptom analysis indicated that GABA-treated plants displayed greater resilience to WBPH infestation compared to untreated plants (Figure 2A). Furthermore, when comparing the symptoms of plants treated with calcium (Ca + WBPH) and those treated with GABA (GABA + WBPH), it was observed that the latter exhibited greater resilience. This increased resilience in GABA + WBPH plants may be attributed to the higher accumulation of GABA and succinate (Figure 5F–I) compared to Ca + WBPH plants, suggesting that GABA-treated plants are more resilient than calcium-treated ones. Further investigations demonstrated that GABA application not only reduced MDA contents (indicative of lipid peroxidation) but also significantly enhanced the activity of antioxidant enzymes such as catalase (CAT), peroxidase (POD), and superoxide dismutase (SOD) under WBPH stress (Figure 3). In response to oxidative stress, plants enhance their antioxidant defense systems by increasing levels of proline, amino acids, anthocyanins, and various enzymes including CAT, POD, SOD, ascorbate peroxidase (APX), and peroxidase (POX). Previous studies have reported that GABA supplementation improves the activity of CAT, POD, and SOD, as well as the total amino acid content (including glutamate, alanine, and proline), while reducing H_2_O_2_ and O_2_^•−^ levels under stress conditions [51,52,53,54]. In line with previous studies, our data suggest that GABA-induced activation of antioxidant pathways enhances the tolerance of rice plants to oxidative stress induced by WBPH infestation. Interestingly, while previous research has primarily focused on abiotic stresses, our findings demonstrate similar results in the context of WBPH stress. Notably, during our screening to determine the optimal level of calcium application, we observed that the number of WBPH was higher in control and 2 mM calcium-supplemented plants compared to those supplemented with 4 mM and 6 mM calcium (Supplementary Figure S2). These results suggest that calcium application may enhance GABA accumulation, which acts as a deterrent to pests such as WBPH. Our findings are supported by previous studies indicating that GABA accumulates rapidly in plants during injury and serves as a deterrent to insect larvae. This suggests a potential mechanism by which calcium supplementation indirectly enhances plant resilience to WBPH infestation through the modulation of GABA levels [55,56,57]. Additionally, a study reported that elevated levels of GABA in Arabidopsis plants inhibited 23% of *Spodoptera littoralis* growth [58]. The increase in GABA levels resulting from mechanical damage can be attributed to elevated Ca^2+^ levels in the targeted and adjacent cells, ultimately triggering glutamate decarboxylase (GAD) enzyme activity [59]. These findings support the concept that systemic GABA regulation in insect invasion may be biologically controlled through Ca^2+^ signaling induced by the GABA shunt pathway. This suggests a sophisticated mechanism by which plants utilize GABA-mediated signaling to deter insect invasion, with calcium playing a pivotal role in orchestrating this response. Further, we noticed that WBPH infestation induced the accumulation of Fe and Mg, while calcium and GABA application enhanced their accumulation against WBPH stress. Fe is a vital element needed for the growth and survival of almost all living organisms. It acts as a catalytic component in various enzymatic processes, including photosynthesis, respiration, and DNA replication [60]. Additionally, Fe is also a component of key antioxidant enzymes involved in detoxification, such as catalase, ascorbate peroxidase, and superoxide dismutase. These enzymes help protect plants from harmful reactive oxygen species (ROS) [61]. When plants are deprived of Fe, they become more vulnerable to chlorosis (yellowing of leaves) and less effective at managing oxidative stress, leading to increased oxidative damage [61]. This makes them more susceptible to stress caused by ROS. Research has shown that supplying iron externally can significantly improve a plant’s ability to tolerate various abiotic stresses, such as drought or high salinity, by enhancing its defense mechanisms against oxidative damage [62]. Our study suggests that GABA and calcium enhances accumulation of Fe to cope with the WBPH-generated oxidative stress. Likewise, Mg is also an important nutrient and essential for plant basic physiological and biochemical processes such as chlorophyll synthesis, transportation, enzyme activation, and protein synthesis [63]. However, there is a lack of research on whether Mg accumulation reduces insect stress; some studies have suggested that Mg application significantly increases the antioxidant defense system during salt stress [64]. Several studies have reported that Mg accumulation not only enhances plant growth but also regulates water efficiency, and induces biotic and abiotic stress tolerance mechanisms [65,66,67]. In tomato, Mg activated the antioxidant defense system against *Ralstonia solanacearum*, and in wheat, it enhanced the activity of photosynthetic machinery and the antioxidant defense system against heat stress [68,69]. These studies and our study suggest that Mg enhances plant tolerance against biotic and abiotic stresses via regulation of the antioxidant defense system.

Our study delves into the intricate regulatory mechanisms of stress hormones, namely ABA and SA, and their role in orchestrating the defense responses of plants against herbivore attacks, particularly focusing on the modulation of stomatal dynamics and relative water content preservation. Under the stress induced by WBPH, we investigated the relationship between ABA and SA levels and the application of calcium and GABA. Our observations revealed a reduction in ABA and SA levels under WBPH stress conditions, whereas plants supplemented with calcium and GABA exhibited significantly higher levels of both hormones compared to control plants and WBPH-infested untreated plants (Figure 4). Additionally, plants treated with Ca + WBPH and GABA + WBPH showed closer stomata (Figure 4A), increased relative water contents (Figure 1H), and higher chlorophyll contents (Figure 6B) compared to WBPH-infested untreated plants. These results suggest that the induction of GABA mediated by calcium enhances water storage through the regulation of ABA-mediated stomatal closure, thereby enhancing photosynthesis efficiency. Notably, our findings contradict previous studies wherein ABA levels were induced in maize attacked by *Diabrotica virgifera virgifera* [70,71], and in Arabidopsis after wounding with *Schistocerca gregaria* [72], as well as where SA levels were induced in tomato infected with potato aphid (*Macrosiphum euphorbiae*) [73]. One potential explanation for this apparent contradiction could stem from prolonged exposure to stress conditions. Extended stress exposure can trigger negative feedback loops or crosstalk with other hormonal pathways, leading to a reduction in ABA and SA levels as a mechanism to prevent excessive signaling. In such cases, the initial induction of ABA and SA in response to stress may be followed by a downregulation to maintain hormonal balance and prevent overactivation of defense responses. This regulatory mechanism ensures that plants allocate resources efficiently and avoid unnecessary energy expenditure on defense signaling. Therefore, the observed decrease in ABA and SA levels under prolonged WBPH stress in our study may reflect a sophisticated adaptive response aimed at optimizing plant survival under sustained herbivore pressure. However, our study found that these hormones were significantly induced in GABA-rich plants under WBPH stress. These results are corroborated by Faraj Hijaz et al. (2012), who demonstrated that the exogenous application of GABA significantly enhances the synthesis of ABA and SA in *Citrus sinensis* compared to control plants [74]. Furthermore, GABA has been shown to promote photosynthetic efficiency by regulating chlorophyll contents and leaf relative water contents in plants such as *Agrostis stolonifera* L. [75], supporting our findings of enhanced ABA levels and stomatal closure under stress conditions. Additionally, GABA induces SA accumulation, enhancing pathogen resilience in plants by regulating antioxidant-related genes, secondary metabolite biosynthesis-related genes, and pathogenesis-related genes [76,77,78]. These findings underscore the multifaceted role of GABA in modulating plant responses to stress, including its involvement in hormonal regulation and defense mechanisms.

In addition to activating the defense system, GABA has been found to enhance various growth parameters in rice under stress conditions, including root/shoot length, leaf width, panicle number and length, and seed development ratio, as illustrated in Figure 1. These findings are consistent with previous research demonstrating that GABA application leads to increased maize root/shoot fresh weight and chlorophyll contents under salt stress, as well as enhanced root/shoot/leaves/stem fresh and dry weight in *Lollo Rosso Lettuce*, and increases shoot/shoot length, root/shoot fresh, and dry weight in mungbean under stress conditions [79,80,81]. Importantly, our study highlights, for the first time, the positive effect of calcium-mediated GABA induction in rice plants against WBPH stress. Nevertheless, it is crucial to note that further research is still needed to fully decipher the mechanism of calcium-induced GABA regulation and activation of defense mechanisms against WBPH stress. These insights underscore the potential of GABA as a key regulator in enhancing plant resilience to various stressors and the importance of understanding its intricate signaling pathways for agricultural applications.

## 5. Conclusions

In conclusion, our study highlights the considerable efficacy of exogenously applied calcium and GABA in enhancing rice plant tolerance to WBPH infestation. Through a comprehensive approach integrating phenotypic assessments and biochemical analyses, we elucidated the intricate mechanisms by which calcium and GABA exert beneficial effects on rice plants under WBPH stress. Our results demonstrate that calcium and GABA supplementation effectively mitigate oxidative stress, modulate stress hormone levels (ABA, SA), enhance antioxidant enzyme activities (CAT, POD, and SOD), and promote the biosynthesis of free amino acids crucial for plant defense. Additionally, these treatments regulate stomatal dynamics, maintain ion homeostasis, and influence the accumulation of key metabolites involved in plant defense mechanisms. Collectively, our findings underscore the potential of exogenous calcium and GABA as potent strategies for enhancing rice plant resilience against WBPH infestation. Nonetheless, further research is essential to elucidate the precise molecular mechanisms underlying these effects and optimize application protocols for practical implementation in rice cultivation. Overall, our study provides valuable insights into innovative approaches for sustainable pest management in rice production systems, with promising implications for improving crop yields and resilience to biotic stressors.

## Data Availability

The data presented in this study are available on request from the corresponding authors.

## Supplementary Materials

The following supporting information can be downloaded at: https://www.mdpi.com/article/10.3390/antiox13111321/s1.

## References

[1] (2017). Faostat. http://www.fao.org/faostat/en/#data.

[2] Prasad R., Shivay Y.S., Kumar D. (2017). Current status, challenges, and opportunities in rice production. Rice Production Worldwide.

[3] Krishnaiah N., Lakshmi V.J., Pasalu I., Katti G., Padmavati C. (2008). Insecticides in Rice IPM: Past, Present and Future.

[4] Cheng X., Wu J., Ma F. (2003). Research and Prevention of the Brown Planthopper.

[5] Dhaliwal G., Arora R. (1994). Trends in Agricultural Insect Pest Management.

[6] Atwal A., Chaudhary J., Sohi B. (1967). Studies on the biology and control of *Sogatella furcifera* Horv.(Delphacidae: Homoptera) in the Punjab. PAU Agric. Res. J..

[7] Salim M., Heinrichs E.A. (1986). Impact of varietal resistance in rice and predation on the mortality of *Sogatella furcifera* (Horvath) (Homoptera: Delphacidae). Crop Prot..

[8] Kumar S., Ram L., Kumar A. (2017). Population dynamics of white backed plant hopper, Sogatella furcifera on basmati rice in relation to biotic and weather parameters. J. Entomol. Zool. Stud..

[9] Ling Y., Weilin Z. (2016). Genetic and biochemical mechanisms of rice resistance to planthopper. Plant Cell Rep..

[10] Steward F.C. (1949). γ-Aminobutyric acid: A constituent of potato tubers?. Science.

[11] Bown A.W., Shelp B.J. (1997). The Metabolism and Functions of [gamma]-Aminobutyric Acid. Plant Physiol..

[12] Ma H. (2003). Plant reproduction: GABA gradient, guidance and growth. Curr. Biol..

[13] Ramesh S.A., Tyerman S.D., Gilliham M., Xu B. (2017). γ-Aminobutyric acid (GABA) signalling in plants. Cell. Mol. Life Sci..

[14] Bown A.W., Shelp B.J. (2016). Plant GABA: Not just a metabolite. Trends Plant Sci..

[15] Kinnersley A.M., Turano F.J. (2000). Gamma aminobutyric acid (GABA) and plant responses to stress. Crit. Rev. Plant Sci..

[16] Li L., Dou N., Zhang H., Wu C. (2021). The versatile GABA in plants. Plant Signal. Behav..

[17] Ansari M.I., Jalil S.U., Ansari S.A., Hasanuzzaman M. (2021). GABA shunt: A key-player in mitigation of ROS during stress. J. Plant Growth Regul..

[18] Suhel M., Husain T., Pandey A., Singh S., Dubey N.K., Prasad S.M., Singh V.P. (2023). An appraisal of ancient molecule GABA in abiotic stress tolerance in plants, and its crosstalk with other signaling molecules. J. Plant Growth Regul..

[19] Molina-Rueda J.J., Pascual M.B., Cánovas F.M., Gallardo F. (2010). Characterization and developmental expression of a glutamate decarboxylase from maritime pine. Planta.

[20] Khan M.I.R., Jalil S.U., Chopra P., Chhillar H., Ferrante A., Khan N.A., Ansari M.I. (2021). Role of GABA in plant growth, development and senescence. Plant Gene.

[21] Carillo P. (2018). GABA shunt in durum wheat. Front. Plant Sci..

[22] Abd El-Gawad H.G., Mukherjee S., Farag R., Abd Elbar O.H., Hikal M., Abou El-Yazied A., Abd Elhady S.A., Helal N., ElKelish A., El Nahhas N. (2021). Exogenous γ-aminobutyric acid (GABA)-induced signaling events and field performance associated with mitigation of drought stress in *Phaseolus vulgaris* L.. J. Plant Signal. Behav..

[23] Yin Y., Yang R., Gu Z. (2014). Calcium regulating growth and GABA metabolism pathways in germinating soybean (*Glycine max* L.) under NaCl stress. Eur. Food Res. Technol..

[24] Feng D., Wang X., Gao J., Zhang C., Liu H., Liu P., Sun X. (2023). Exogenous calcium: Its mechanisms and research advances involved in plant stress tolerance. Front. Plant Sci..

[25] Sun X., Pan B., Wang Y., Xu W., Zhang S. (2020). Exogenous calcium improved resistance to Botryosphaeria dothidea by increasing autophagy activity and salicylic acid level in pear. Mol. Plant-Microbe Interact..

[26] Aldon D., Mbengue M., Mazars C., Galaud J.-P. (2018). Calcium signalling in plant biotic interactions. Int. J. Mol. Sci..

[27] Taiz L., Zeiger E. (2010). Plant Physiology.

[28] Maathuis F.J.M. (2009). Physiological functions of mineral macronutrients. Curr. Opin. Plant Biol..

[29] Lecourieux D., Ranjeva R., Pugin A. (2006). Calcium in plant defence-signalling pathways. New Phytol..

[30] Bai Q., Yang R., Zhang L., Gu Z. (2013). Salt stress induces accumulation of γ–aminobutyric acid in germinated foxtail millet (*Setaria italica* L.). Cereal Chem..

[31] Li J., Zhou Q., Zhou X., Wei B., Zhao Y., Ji S. (2020). Calcium treatment alleviates pericarp browning of ‘nanguo’pears by regulating the GABA shunt after cold storage. Front. Plant Sci..

[32] Zhao D.-D., Jang Y.-H., Kim E.-G., Park J.-R., Jan R., Lubna, Asaf S., Asif S., Farooq M., Chung H. (2023). Identification of a Major Locus for Lodging Resistance to Typhoons Using QTL Analysis in Rice. Plants.

[33] Jan R., Kim N., Asaf S., Lubna, Asif S., Du X.X., Kim E.G., Jang Y.H., Kim K.M. (2023). OsCM regulates rice defence system in response to UV light supplemented with drought stress. Plant Biol..

[34] Jan R., Asif S., Asaf S., Lubna, Khan Z., Khan W., Kim K.-M. (2024). Gamma-aminobutyric acid treatment promotes resistance against Sogatella furcifera in rice. Front. Plant Sci..

[35] Jan R., Kim N., Lee S.-H., Khan M.A., Asaf S., Park J.-R., Asif S., Lee I.-J., Kim K.-M. (2021). Enhanced flavonoid accumulation reduces combined salt and heat stress through regulation of transcriptional and hormonal mechanisms. Front. Plant Sci..

[36] Chao Y.-Y., Chen C.-Y., Huang W.-D., Kao C.H. (2010). Salicylic acid-mediated hydrogen peroxide accumulation and protection against Cd toxicity in rice leaves. Plant Soil.

[37] Patterson B.D., MacRae E.A., Ferguson I.B. (1984). Estimation of hydrogen peroxide in plant extracts using titanium (IV). Anal. Biochem..

[38] Elstner E.F., Heupel A. (1976). Inhibition of nitrite formation from hydroxylammoniumchloride: A simple assay for superoxide dismutase. Anal. Biochem..

[39] Johansson L.H., Borg L.H. (1988). A spectrophotometric method for determination of catalase activity in small tissue samples. Anal. Biochem..

[40] Khan Z., Jan R., Asif S., Farooq M., Jang Y.-H., Kim E.-G., Kim N., Kim K.-M. (2024). Exogenous melatonin induces salt and drought stress tolerance in rice by promoting plant growth and defense system. Sci. Rep..

[41] Lubna, Khan M.A., Asaf S., Jan R., Waqas M., Kim K.-M., Lee I.-J. (2022). Endophytic fungus *Bipolaris* sp. CSL-1 induces salt tolerance in *Glycine max.* L via modulating its endogenous hormones, antioxidative system and gene expression. J. Plant Interact..

[42] Jan R., Khan M.A., Asaf S., Lubna, Lee I.-J., Kim K.-M. (2021). Over-expression of chorismate mutase enhances the accumulation of salicylic acid, lignin, and antioxidants in response to the white-backed planthopper in rice plants. Antioxidants.

[43] Weckwerth W., Wenzel K., Fiehn O. (2004). Process for the integrated extraction, identification and quantification of metabolites, proteins and RNA to reveal their co-regulation in biochemical networks. Proteomics.

[44] Sobolevsky T.G., Revelsky A.I., Miller B., Oriedo V., Chernetsova E.S., Revelsky I.A. (2003). Comparison of silylation and esterification/acylation procedures in GC-MS analysis of amino acids. J. Sep. Sci..

[45] Jan R., Khan M.-A., Asaf S., Waqas M., Park J.-R., Asif S., Kim N., Lee I.-J., Kim K.-M. (2022). Drought and UV radiation stress tolerance in rice is improved by overaccumulation of non-enzymatic antioxidant flavonoids. Antioxidants.

[46] Pavlík M., Pavlíková D., Zemanová V., Hnilička F., Urbanová V., Száková J. (2012). Trace elements present in airborne particulate matter—Stressors of plant metabolism. Ecotoxicol. Environ. Saf..

[47] Sita K., Kumar V. (2020). Role of Gamma Amino Butyric Acid (GABA) against abiotic stress tolerance in legumes: A review. Plant Physiol. Rep..

[48] Mei X., Chen Y., Zhang L., Fu X., Wei Q., Grierson D., Zhou Y., Huang Y., Dong F., Yang Z. (2016). Dual mechanisms regulating glutamate decarboxylases and accumulation of gamma-aminobutyric acid in tea (*Camellia sinensis*) leaves exposed to multiple stresses. Sci. Rep..

[49] Bhattacharya S., Khatri A., Swanger S.A., DiRaddo J.O., Yi F., Hansen K.B., Yuan H., Traynelis S.F. (2018). Triheteromeric GluN1/GluN2A/GluN2C NMDARs with unique single-channel properties are the dominant receptor population in cerebellar granule cells. Neuron.

[50] Kiep V., Vadassery J., Lattke J., Maaß J.P., Boland W., Peiter E., Mithöfer A. (2015). Systemic cytosolic Ca^2+^ elevation is activated upon wounding and herbivory in Arabidopsis. New Phytol..

[51] Hasan M.M., Alabdallah N.M., Alharbi B.M., Waseem M., Yao G., Liu X.-D., Abd El-Gawad H.G., El-Yazied A.A., Ibrahim M.F., Jahan M.S. (2021). GABA: A key player in drought stress resistance in plants. Int. J. Mol. Sci..

[52] Dabravolski S.A., Isayenkov S.V. (2023). The role of the γ-aminobutyric acid (GABA) in plant salt stress tolerance. Horticulturae.

[53] Zhang L., Becker D. (2015). Connecting proline metabolism and signaling pathways in plant senescence. Front. Plant Sci..

[54] Suzuki N., Mittler R. (2006). Reactive oxygen species and temperature stresses: A delicate balance between signaling and destruction. Physiol. Plant..

[55] Wallace W., Secor J., Schrader L.E. (1984). Rapid accumulation of γ-aminobutyric acid and alanine in soybean leaves in response to an abrupt transfer to lower temperature, darkness, or mechanical manipulation. Plant Physiol..

[56] Ramputh A.-I., Bown A.W. (1996). Rapid [gamma]-aminobutyric acid synthesis and the inhibition of the growth and development of oblique-banded leaf-roller larvae. Plant Physiol..

[57] Bown A.W., Hall D.E., MacGregor K.B. (2002). Insect footsteps on leaves stimulate the accumulation of 4-aminobutyrate and can be visualized through increased chlorophyll fluorescence and superoxide production. Plant Physiol..

[58] Scholz S.S., Reichelt M., Mekonnen D.W., Ludewig F., Mithöfer A. (2015). Insect herbivory-elicited GABA accumulation in plants is a wound-induced, direct, systemic, and jasmonate-independent defense response. Front. Plant Sci..

[59] Seifikalhor M., Aliniaeifard S., Hassani B., Niknam V., Lastochkina O. (2019). Diverse role of γ-aminobutyric acid in dynamic plant cell responses. Plant Cell Rep..

[60] Pandey S.S. (2023). The Role of Iron in Phytopathogenic Microbe–Plant Interactions: Insights into Virulence and Host Immune Response. Plants.

[61] Rai S., Singh P.K., Mankotia S., Swain J., Satbhai S.B. (2021). Iron homeostasis in plants and its crosstalk with copper, zinc, and manganese. Plant Stress.

[62] Tripathi D.K., Singh S., Gaur S., Singh S., Yadav V., Liu S., Singh V.P., Sharma S., Srivastava P., Prasad S.M. (2018). Acquisition and homeostasis of iron in higher plants and their probable role in abiotic stress tolerance. Front. Environ. Sci..

[63] Ishfaq M., Wang Y., Yan M., Wang Z., Wu L., Li C., Li X. (2022). Physiological essence of magnesium in plants and its widespread deficiency in the farming system of China. Front. Plant Sci..

[64] Abd El-Mageed T.A., Gyushi M.A., Hemida K.A., El-Saadony M.T., Abd El-Mageed S.A., Abdalla H., AbuQamar S.F., El-Tarabily K.A., Abdelkhalik A. (2022). Coapplication of effective microorganisms and nanomagnesium boosts the agronomic, physio-biochemical, osmolytes, and antioxidants defenses against salt stress in Ipomoea batatas. Front. Plant Sci..

[65] Senbayram M., Gransee A., Wahle V., Thiel H. (2015). Role of magnesium fertilisers in agriculture: Plant–soil continuum. Crop Pasture Sci..

[66] Rodrigues V.A., Crusciol C.A.C., Bossolani J.W., Moretti L.G., Portugal J.R., Mundt T.T., de Oliveira S.L., Garcia A., Calonego J.C., Lollato R.P. (2021). Magnesium foliar supplementation increases grain yield of soybean and maize by improving photosynthetic carbon metabolism and antioxidant metabolism. Plants.

[67] Chen Z.C., Peng W.T., Li J., Liao H. (2018). Functional dissection and transport mechanism of magnesium in plants. Semin. Cell Dev. Biol..

[68] Imada K., Sakai S., Kajihara H., Tanaka S., Ito S. (2016). Magnesium oxide nanoparticles induce systemic resistance in tomato against bacterial wilt disease. Plant Pathol..

[69] Mengutay M., Ceylan Y., Kutman U.B., Cakmak I. (2013). Adequate magnesium nutrition mitigates adverse effects of heat stress on maize and wheat. Plant Soil.

[70] Erb M., Flors V., Karlen D., De Lange E., Planchamp C., D’Alessandro M., Turlings T.C., Ton J. (2009). Signal signature of aboveground-induced resistance upon belowground herbivory in maize. Plant J..

[71] Forterre Y., Skotheim J.M., Dumais J., Mahadevan L. (2005). How the Venus flytrap snaps. Nature.

[72] Schäfer M., Fischer C., Meldau S., Seebald E., Oelmüller R., Baldwin I.T. (2011). Lipase activity in insect oral secretions mediates defense responses in Arabidopsis. Plant Physiol..

[73] Li Q., Xie Q.-G., Smith-Becker J., Navarre D.A., Kaloshian I. (2006). Mi-1-mediated aphid resistance involves salicylic acid and mitogen-activated protein kinase signaling cascades. Mol. Plant-Microbe Interact..

[74] Hijaz F., Nehela Y., Killiny N. (2018). Application of gamma-aminobutyric acid increased the level of phytohormones in *Citrus sinensis*. Planta.

[75] Li Z., Cheng B., Peng Y., Zhang Y. (2020). Adaptability to abiotic stress regulated by γ-aminobutyric acid in relation to alterations of endogenous polyamines and organic metabolites in creeping bentgrass. Plant Physiol. Biochem..

[76] Rasool N. (2022). Plant hormones: Role in alleviating biotic stress. Plant Horm. Recent Adv. New Perspect. Appl..

[77] Gao Q.-M., Zhu S., Kachroo P., Kachroo A. (2015). Signal regulators of systemic acquired resistance. Front. Plant Sci..

[78] Tada Y., Spoel S.H., Pajerowska-Mukhtar K., Mou Z., Song J., Wang C., Zuo J., Dong X. (2008). Plant immunity requires conformational charges of NPR1 via S-nitrosylation and thioredoxins. Science.

[79] Aljuaid B.S., Ashour H. (2022). Exogenous γ-aminobutyric acid (GABA) application mitigates salinity stress in maize plants. Life.

[80] Mirzaei S., Moradi S., Karimi M., Esmaeili S., Gruda N.S., Aliniaeifard S. (2024). Gamma-Aminobutyric Acid-Mediated Alkalinity Stress Alleviation in Lollo Rosso Lettuce under Diverse Light Spectra. Agronomy.

[81] Ullah A., Ali I., Noor J., Asghar M.A., Javed H.H., Ullah S. (2023). Exogenous γ-aminobutyric acid (GABA) mitigated salinity-induced impairments in mungbean plants by regulating their nitrogen metabolism and antioxidant potential. Front. Plant Sci..

