# Supplementary material for: Augmenting Rice Defenses: Exogenous Calcium Elevates GABA Levels Against WBPH Infestation
Source: Antioxidants (Basel). 2024 Oct 30;13(11):1321. doi: 10.3390/antiox13111321 (PMC11591109; doi:10.3390/antiox13111321)
Supplement: Supplementary file 1 [file antioxidants-13-01321-s001.zip › antioxidants-3198436-supplementary.pdf]

Augmenting Rice Defenses: Exogenous Calcium Elevates GABA Levels against WBPH Infestation

Rahmatullah Jan1[,2,*], Sajjad Asaf[3], Lubna[3], Muhammad Farooq[4], Saleem Asif[1], Zakirullah Khan[1], Kyung-Min Kim[1,2,*]

1Department of Applied Biosciences, Graduate School, Kyungpook National University, Daegu, 41566, South Korea

2Coastal Agriculture Research Institute, Kyungpook National University, Daegu, 41566, South Korea

3Natural and Medical Science Research Center, University of Nizwa, Nizwa, Oman

4Department of Agriculture Biology, College of Agriculture and Life Sciences, Jeonbuk National University, Jeonju Korea

* Correspondence: kkm@knu.ac.kr +82-53-950-5711; rehmatbot@yahoo.com +82-10-54750315

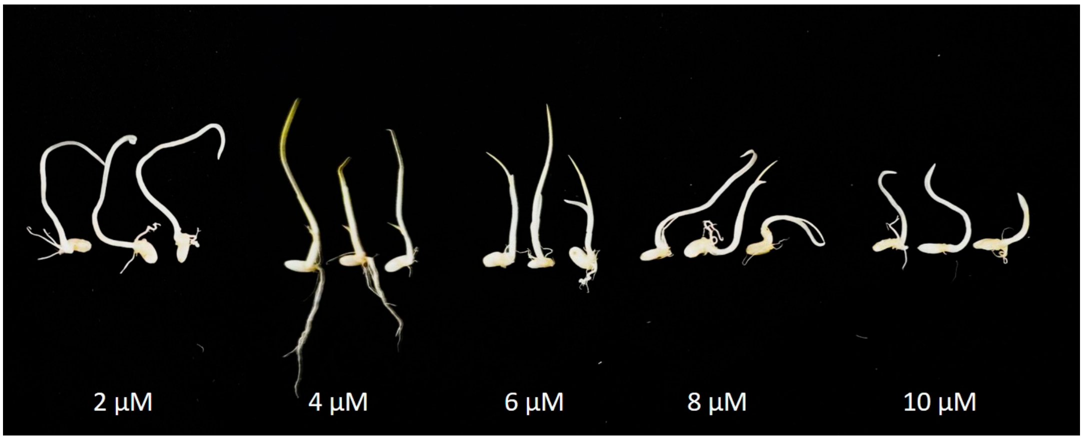

Supplementary Figure S1. Screening of rice seed growth pattern under varying concentrations of calcium.

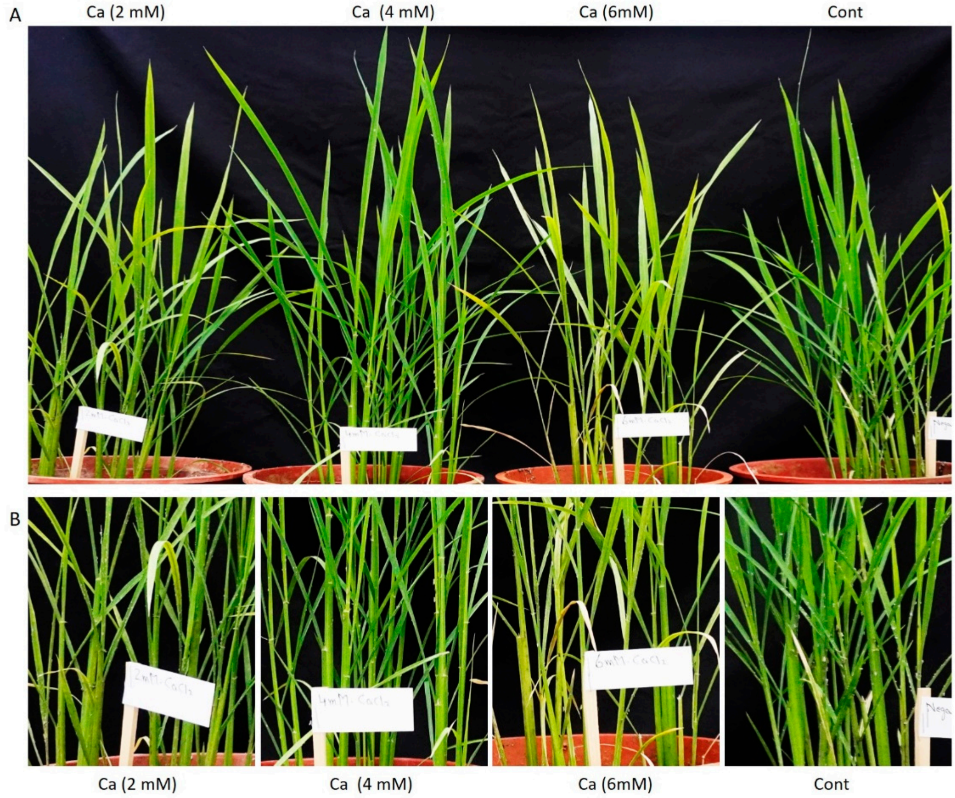

Supplementary Figure S2. Panel **A** presents images of whole rice plants subjected to WBPH attack under different calcium concentrations. Panel **B** provides a magnified view showing the WBPH population feeding on the rice plants, highlighting the impact of varying calcium concentrations on WBPH population efficiency.
